# Exploring Learning Engagement in Rural and Urban Nursing Placements: A Five-Year Mixed-Methods Study

**DOI:** 10.3390/ijerph23020163

**Published:** 2026-01-28

**Authors:** Sandra Coe, Annette Marlow, Sarah J. Prior, Carey Mather

**Affiliations:** 1 Rural Clinical School, University of Tasmania, Burnie, TAS 7320, Australia; 2 Tasmanian School of Medicine, University of Tasmania, Hobart, TAS 7000, Australia; annettemarlow23@gmail.com; 3 Tasmanian School of Medicine, University of Tasmania, Burnie, TAS 7320, Australia; 4 School of Nursing, Paramedicine and Healthcare Sciences, Charles Sturt University, Wagga Wagga, NSW 2678, Australia; carey.mather@utas.edu.au; 5 School of Nursing, University of Tasmania, Launceston, TAS 7250, Australia

**Keywords:** nursing placement, professional experience, student experience, rural placement

## Abstract

Professional experience placements are a requirement for undergraduate nursing students enabling real world skill development. Barriers to meaningful and positive placements have previously been reported, however there is limited research on how the location of placement impacts the student experience and outcomes. This study investigates the placement experiences of undergraduate nursing students at the University of Tasmania (UTAS) over a five-year period, with a focus on urban versus rural settings and year-level differences. Findings reveal that over one-third of students reported constructive placement experiences, with rural placements yielding slightly more positive outcomes than urban ones. First-year students were more likely to report constructive experiences compared to their senior counterparts, suggesting that longer placement durations may contribute to increased dissatisfaction. Quality of placement—defined by supervision and skill development—emerged as the most influential factor in shaping student experiences. While most students praised the quality of supervision, third-year students expressed both the highest praise and criticism. Opportunities for clinical and interpersonal skill development were central to students’ perceptions of placement quality, with rural placements slightly outperforming urban in skill development. However, some students, particularly in later years, felt that certain venues lacked adequate opportunities for skill acquisition. The study underscores the importance of high-quality supervision and appropriate clinical settings in enhancing placement experiences and suggests that constructive placements are more conducive to learning. These insights can inform strategies to improve the educational value of nursing placements across diverse settings.

## 1. Introduction

Work integrated learning for undergraduate nursing students in Australia is known as professional experience placements (PEP) (‘placements’) and are a compulsory feature of undergraduate nurse educational preparation [1]. Placements enable real world learning that stimulates skills development and immerses students in the practical aspects of the profession to develop the requisite capabilities defined by the Australian Nursing and Midwifery Accreditation Council (ANMAC) Standards (2019) [1]. Nursing placement has been shown to create significant personal development opportunities with increased appreciation and sensitivity toward cultural issues and cross-cultural care [2], professional socialisation, exposure to different clinical areas [3], and clearer career planning [4]. There is enduring national and international interest examining what constitutes effective placement learning experiences. Three overarching categories define the focus of much of this research—student experience, supervision, and setting [5]. Supervision, which includes staff attitudes, and interactions between educators and students, appears to be the most reported factor when it comes to student satisfaction with their placements [5,6]. The student experience of placements has been reported as involving a wide range of emotional, professional, and interpersonal challenges that shape the overall journey. These include increased pride, self-confidence, and a desire to make a difference [7] as well as insecurity, a reality shock, and a need for support and guidance [7,8]. Furthermore, the clinical environment and the service type were also found to be fundamental influencing factors in placement satisfaction in an EU study [6], consistent with an Australian study that suggested decreased placement satisfaction when a lack of support was available [9]. It has been shown globally that overall nursing student satisfaction is strongly related to their placement experience and has a significant impact on student retention [10,11,12,13]. Previous research has identified a need to improve nursing placements, with a focus on delivering consistent, high-quality programmes, understanding barriers and enablers to student learning, and enhancing student outcomes [14]. Evidence from the United Kingdom highlights three characteristics of high-quality placements: students feeling part of the team, receiving adequate support to build confidence and competence, and having their anxiety and initial lack of preparedness acknowledged [15]. In the Australian context, three key enablers of positive placement experiences have been identified: enjoyment of place (“environment and community”), a welcoming and responsive workplace (“friendly and supportive workplace”), and diverse learning opportunities (“exposure to broad practice”) [16]. Conversely, five common barriers have been reported, including financial pressures, relocation challenges, disruption to personal routines, stress, and communication difficulties [16]. A review of student narratives on placement supervision further emphasises the importance of supportive instructors, close supervision, and a sense of belonging, while unsupportive behaviours and inadequate supervision diminish placement quality [17].

Given the ongoing challenges in nursing workforce capacity, understanding what contributes to constructive placement experiences is essential for quality assurance and reducing student attrition. This is particularly critical in rural areas, where workforce shortages significantly affect service accessibility and delivery [18]. Prior research has shown that positive rural placements can influence graduates’ decisions to remain in and work in rural settings [18,19,20]. Furthermore, Experiential Learning Theory (ELT) [21,22] emphasises the impact of cognitive, environmental, and emotional experiences in the learning process and outcomes. ELT suggests that learners develop skills more effectively when they engage with authentic, real-world contexts, such as those provided through work-integrated learning. This is particularly relevant in rural and regional settings, where learners are exposed to broader scopes of practice, closer clinician–community relationships, and the unique challenges associated with limited resources and workforce shortages. Such environments provide rich, immersive learning opportunities that can accelerate skill development and foster adaptability, resilience, and a deeper understanding of rural health contexts.

Despite sustained interest in professional experience placements (PEP), few studies have directly compared the experiences of students undertaking urban placements with those completing rural placements. Even fewer have used student-generated narratives to explore how learners themselves describe and construct effective placement experiences. We aim to answer the following research questions.

1. How do professional experience placement experiences differ for student nurses in urban versus rural healthcare settings?
2. How do learning opportunities and support structures for student nurses vary between urban and rural placement settings?
3. How do urban and rural professional experience placements shape student nurses’ perceptions of preparedness for professional practice?

## 2. Materials and Methods

The study involved a comprehensive mixed methods analysis of narrative feedback from nursing students at all levels of undergraduate nursing at an Australian University. The dataset consisted of reflective feedback submitted as part of routine placement evaluations, and survey responses in the form of checkbox answers, sliding scales, and Likert scale responses. A mixed-methods design was chosen to capture both the breadth and depth of students’ experiences. The decision to employ a mixed-methods design is supported by Creswell’s widely cited work on mixed-methods research [23], which emphasises the value of integrating quantitative and qualitative approaches to gain a more comprehensive understanding of complex educational and social phenomena. Creswell argues that mixed-methods designs are particularly appropriate when neither quantitative nor qualitative data alone can adequately capture the full scope of the research problem. In this study, learning engagement during professional experience placements is influenced by contextual, relational, and experiential factors that benefit from both measurable patterns and rich narrative insight. The mixed-methods approach strengthened this study by combining measurable trends with in-depth insights into students’ lived experiences.

### 2.1. Setting

The study explores the narrative experiences of University of Tasmania (UTAS) undergraduate nursing students. UTAS is the only university located in Tasmania (an island state of Australia). The Modified Monash Model (MMM) is a geographical location classification system used by the Australian Government to determine the rurality of a region [24]. Tasmania has two main urban centres, approximately 200 km apart. The remainder of the state is classified as regional, rural, remote and very remote. Nursing students at UTAS study across four primary campuses (Table 1). Student placements in Tasmania occur across Tasmania and include remote island regions. Student placements in New South Wales (NSW) primarily occur in Sydney (the state’s capital city), although rural placements in NSW occurred in the later years of this study. During the period of this study, UTAS nursing students undertook five placements across their pre-registration degree programme—a two-week placement in the first-year, two three-week placements in second year, and a six- and seven-week placement in third year [25]. During nursing placements, there are a variety of methods of support and supervision available to students [26], which are related to geographic location, formal placement agreement/s and health service provider requirements. Students are also supervised by staff within placement organisations, who perform this supervision as part of their clinical role. The term ‘rural’ in this paper is used to represent regional rural, remote, and very remote regions as classified by the MMM [24].

### 2.2. Data Collection

This study utilised secondary text data collected as part of a routine administrative survey distributed to all nursing students at the end of every nursing placement by the UTAS College of Health and Medicine—the Placement survey. This survey is accessed by students via the Placement Management System as part of their placement completion tasks and is an optional feedback mechanism. Students complete and submit the survey online.

The survey data covered the period 1 January 2017 to 31 December 2021, and the survey questions remained unchanged across the five-year data collection period. All nursing students were invited to complete the survey regardless of where placement occurred. The survey contained demographic questions about year of study, unit code, and placement facility, and three questions inviting free-text feedback on placement experiences:

1. Please list up to 3 aspects of this professional experience placement that have helped your learning.
2. Please list up to 3 aspects of this professional experience placement that have hindered your learning.
3. Are there any additional comments or suggestions you would like to make to improve your PEP learning experience?

### 2.3. Data Analysis

Initially, all data were descriptively coded to the demographic variables of the year of survey, student’s year of study, and placement state. All other feedback data was analysed using content analysis with an inductive approach to enable interpretation using a “systematic classification process of coding and identifying themes or patterns” [27]. Data from each feedback question was manually coded with content variables created inductively. Following the identification of the main variables, categories were identified, and comparative analyses were undertaken between sub-groups within each category. Trends and patterns were documented and quantified for reporting purposes [28]. Content analysis was an appropriate method for this research to investigate the primary categories in the data and record trends over the five-year research period.

The content analysis was guided by three questions.

1. What helped and what hindered nursing students’ learning during placements?
2. Did these factors change as students progressed through their degree?
3. Did these factors change by placement region?

Data analysis occurred using Microsoft Excel, enabling the calculation of responses for each variable and comparisons made between student sub-groups. These calculations are presented as percentages.

Several strategies were employed to minimise bias during the categorisation and interpretation of qualitative data. An inductive content-analysis approach was used to allow categories and themes to emerge from the data rather than being shaped by predetermined assumptions. To enhance the credibility of the coding process, an initial subset of responses was independently coded by two researchers. Coding decisions were then compared and discussed, with discrepancies resolved through consensus. This process supported consistency in the application of codes and reduced the influence of individual researcher perspectives. Reflexive practices were incorporated throughout the analysis, including regular team discussions to review emerging categories.

These strategies collectively strengthened the trustworthiness of the analysis and helped ensure that the categorisation of qualitative data was systematic, transparent, and as free from bias as possible.

The project received ethics approval from the UTAS Human Research Ethics Committee (H0026757) and is funded under the Rural Health Multidisciplinary Training (RHMT) project. Generative Artificial Intelligence (GenAI) was not used in any part of this study.

## 3. Results

### 3.1. Demographic Data

The research team received 3081 responses for the *Placement* survey for the five-year period. Responses increased each year, except in 2020, and students from 2021 were over-represented in the research results (27%). Rural NSW placements represented 1% of the overall results (Table 2).

Second year students (41%) were over-represented in the study, followed by third year students (35%), with first-year students under-represented (24%). Second- and third-year students each had two placements a year, with first-year students having one. Potentially, second- and third-year students completed the survey twice a single year, which may account for the higher response rates for these student year groups. Students with urban placements were over-represented in the results by a ratio of 3:1 (76% versus rural placements 24%). Placements in Hobart were also over-represented (34%), with Sydney placements second highest (25%), followed by rural TAS (22%). Placements in Launceston (17%) and rural NSW were under-represented (1%). Rural placements in NSW were limited during the study period accounting for this low percentage.

Placement supervision was consistent in the student narratives with praise for supervisors having the highest response rate (53%) and supervision criticism third highest (31%). Developing clinical skills was second highest (43%), and developing interpersonal skills was fourth (26%).

### 3.2. Categories

Three overarching categories emerged from the data: Overall placement experience, supervision during placement, and skills development. Table 3 shows the coding variables for each category. Each category is further described below.

#### 3.2.1. Category 1: Overall Placement Experience

The results suggest most students had constructive placements during the study period (Table 4). Almost a fifth of students (19%) indicated nothing hindered their learning and 15% indicated they had constructive placements. Only 2% indicated nothing helped their learning during placement.

A higher percentage of rural placement students (17%) indicated they had constructive placements than those with urban placements (15%), and slightly more students with urban placements (20%) indicated their learning was not hindered during placement than those with rural placements (19%).

Fewer first-year students indicated nothing helped learning (1%), compared with second (2%) and third year students (3%). Fewer second-year students (17%) indicated nothing hindered learning compared with first- and third-year students (21%). More first-year students had a constructive placement experience (18%) compared with second- (15%) and third-year students (14%). Table 5 highlights student narratives about the quality of their experiences coded to these variables.

Most of the comments about ‘nothing helping learning’ came from second- and third-year students in 2017. Some students asserted a lack of learning and/or unhelpful placement experience, with claims the placement was ‘chaotic’ or caused students’ stress’. Students that had constructive and helpful learning experiences appeared to reject the notion that their learning was hindered during placement and emphasised the positive experience and learning value of the placement.

#### 3.2.2. Category 2: Placement Supervision

Some students specified a difference between the UTAS funded supervisors, and the supervisors within the placement organisation (Table 6). When no differentiation was specified in the narrative, it was coded as supervisors within the placement organisation, although it is acknowledged this may have inflated the responses for this supervisor group. More than half (60%) of the student narratives praised their supervision during placement (some praising both groups of supervisors). Around a third criticised their supervision (37%). There were higher rates of praise (53%) and criticism (31%) for placement preceptors, supervisors, and staff (placement supervision) compared with university funded facilitators (UFFs) (20% praise and 7% criticism). Although this may be due to coding as explained above. Praise for UFFs was higher for urban placements (23%) compared to rural placements (11%). Third-year students offered more praise for UFFs (26%) than second- (19%) and first-year students (14%). Praise for placement supervisors was higher for rural placements (55%) compared to urban placements (52%), and again, third-year students offered more praise (55%), than first- (53%) and second-year students (50%).

Overall, supervision criticism for UFFs was low and first-year students had the lowest percentage of criticism (3%), and third-year students had the highest (9%). Criticism for placement supervision was lower for third-year students (30%), with rural placements the lowest (29%). First-year students had low rates of supervision criticism for UFFs, although their supervision criticisms of placement supervision were equal with second-year students, urban placements, and the combined percentage. Student narratives about supervision are shown in Table 7.

Student praise for UFFs included making time to work with students, providing support and resources. Supervision criticisms asserted a lack of support, being unapproachable, not spending enough time with students, or an absence of UFFs altogether. Some students felt disempowered by UFFs with comments that indicated this factor impacted their wellbeing.

Student praise for placement supervision indicated some staff were welcoming and supportive, available for teaching, and demonstrated patience with students. Supervision criticisms asserted placement staff sometimes did not want to teach students, did not allow students to undertake specific tasks, were rude, unsupportive, and lacked awareness of student capabilities. Some students indicated placement facilities were short-staffed or lacked registered nurses, with both impacting the quality of placement supervision available to students.

#### 3.2.3. Category 3: Skills Development

Results indicated skills development was an important aspect for students for their placement experience (see Table 8). Students asserted that their development of clinical and interpersonal skills during placement was an important outcome of their experiences. Within this theme, developing clinical skills (43%) ranked higher than the development of interpersonal skills (26%). Student criticisms expressed a lack of opportunity to develop clinical skills (24%), with requests for more skills development opportunities during placements (8%). Some students indicated scope of practice limitations also impacted their placement experiences (7%), and some asserted acute settings were necessary for skills development during placement (4%).

Developing interpersonal skills was higher for urban placements (28%) than it was for rural placements (21%). Developing clinical skills was higher for rural areas (45%) than it was for urban placements (42%). Assertions there were limited opportunities to develop clinical skills during placement were higher for rural areas (26%) than it was for urban placements (23%). Development of clinical skills appeared to decrease as students progressed through their degree programme. First-year students had the highest rate (50%), followed by second- (44%) and third-year students (36%). First-year students also had the highest rate for developing interpersonal skills (31%), followed by third- (26%) and second-year students (24%). More second-year students asserted placements provided limited opportunities to develop clinical skills (26%) compared to first- and third-year students (22%).

More first-year students asserted scope of practice restrictions limited skills development (18%) compared to second- (4%) and third-year students (2%). Second- and third-year students asserted the need for more opportunities to develop skills (9%), and the need for acute settings for skills development (6% and 4%). Table 9 highlights student narratives about skills development during placements.

Student comments about limited opportunities to develop clinical skills often arose alongside criticisms of placement guidance, such as assertions that facility staff were too busy, did not want to teach students, lack of registered nurse (RN) supervision, or facilities being too quiet or unsuitable for the student’s learning level. Student comments about the scope of practice restrictions argued it hindered their learning opportunities, although some suggested placement supervisors expected students to undertake tasks beyond their scope of practice. Student comments that asserted the need for more opportunities to develop clinical skills also arose alongside comments about facility staff being too busy or not wanting to teach students. Student comments that argued for placements to occur in acute clinical settings were generally from second- and third-year students and often asserted the current placement facility was more suitable for earlier year students.

## 4. Discussion

This study aimed to explore and understand the experiences of undergraduate nursing students during placements. Overall, more than one third of nursing students in this study indicated they had constructive placement experiences with students undertaking rural placements having more constructive placements than their urban counterparts. Stratified results indicated more first-year students had constructive placements than second- and third-year students. Potentially the longer placement periods for second- and third-year students increased the opportunities for dissatisfaction with the overall experience. These findings are at odds with those from the previous rapid review [5] that found that most students in their review studies reported clinical placements as “stressful and overwhelming”, suggesting they were unhelpful experiences.

Our findings indicate that quality was the primary variable impacting students’ placement experiences, with ‘quality’ comprising supervision and skills development, irrespective of whether supervision was by placement staff or UFFs. These findings parallel other research that found “supervisory relationship(s)” to be “key to students learning” during placements [5,17]. Supervision emerged as a critical element of students’ experiences which accords with other research where Currie, Grootemaat, Samsa et al. assert multiple factors impact students’ placement experiences, with their “perception(s) of the attitude of the clinical staff they are working with” “one of the most significant” [5]. Most students praised the quality of supervision during placement, with this rate unchanged with urban or rural placements. One-third of students criticised the quality of supervision, and this rate increased slightly for urban placements, and reduced for rural placements. This suggests, contrary to other research, that UTAS students on rural placements expressed slightly more satisfaction with their supervision during placement than those with urban placements. It may be that the Whole of Community Facilitator Model of support provided by UTAS for rural placements increases this level of support [26]. The rate of praise and criticism for placement supervision also varied by students’ year of study. Third-year students were highest in both their praise and criticism of supervision during placement, followed by second-year students, with first-year students having the lowest rate for both categories.

Unsurprisingly, learning was also a significant factor in quality placements with opportunities to develop clinical and interpersonal skills during placement dominant in student narratives in this study. More students asserted they developed skills (clinical (43%) and interpersonal (26%)) than a lack of opportunities (24%). This result supports the finding that, overall, most UTAS nursing students experienced constructive placements during the five-year analysis period.

When these results were stratified by placement type there was a minimal difference between urban and rural placements. Students who felt they developed clinical skills during rural placements were slightly higher (45%) than urban placements (42%), and higher for first year (50%) than second (44%) or third-year students (36%). Students who believed they developed interpersonal skills were higher for urban placements (28%) and rural (21%). First-year students were also highest in this category (31%) compared o third (26%) and second-year students (24%).

More students with rural placements asserted a lack of opportunity to develop clinical skills (26%) than students with urban placements (23%). Given the importance students in this study placed on developing skills during placement, it is unsurprising some students stressed the importance that placements occur in acute clinical settings to develop clinical skills, particularly for later-year students. Some placement venues were considered ‘inappropriate’ for skills development for the later year students. These findings are in accordance with other studies that found students value hospital settings as critical learning spaces [17,29,30].

It is acknowledged that the research explores students’ perceptions of their experiences only, and that student enjoyment is not a measure of actual learning that may have occurred during placement.

The findings from this study indicate a constructive placement experience is more inducive to learning than a challenging one. As students’ progress through their degree programme, the findings indicate they may become more critical of their placement experiences as second- and third-year students have longer placements than first-year students. Longer placements may produce challenges due to the length of exposure to a single placement facility. In contrast to other research, the results indicate relatively little difference between students’ urban and rural placement experiences [31,32,33,34]. The results from this study add to a broader understanding of students’ placement experiences and the findings can inform quality improvement practices to enhance the learning potential of nursing student placements.

Recommendations from this study include aligning placement settings with nursing students’ year level to ensure developmental appropriateness and progressive skill acquisition. Prioritising the quality of supervision is essential, as effective clinical teaching and consistent preceptor engagement are critical determinants of students’ learning, confidence, and professional identity formation. The findings also highlight the need to identify placement environments that may be unsuitable for advanced-level students, particularly where the scope of practice, case complexity, or supervisory capacity does not support higher-level capability development.

Further recommendations include strengthening partnerships between education providers and clinical agencies to ensure shared expectations regarding student capability, supervision standards, and assessment processes. Enhanced monitoring of placement quality, including systematic feedback from students and supervisors, would support continuous improvement and early identification of environments requiring additional support or remediation. Finally, future research should explore the structural and contextual factors that influence placement suitability across different settings, with the aim of informing evidence-based placement allocation frameworks that optimise learning outcomes and workforce readiness.

### Limitations

This study included experiences of students from only one university and may not be generalisable to other higher education institutions. This study also relied on self-reported data which may not accurately reflect the actual learning outcomes or clinical competence. Although this data was captured over a five-year period, there is no longitudinal follow up which may limit the insights into how placement experiences evolve over time. In addition, the survey did not assess factors such as student stress levels or the availability of social support during placements. These variables are known to influence learning engagement and overall placement experience, and their absence restricts our ability to understand how psychosocial factors may have shaped the reported outcomes.

## 5. Conclusions

Undergraduate nursing student placement experiences differ across geographical regions; however, the concepts that influence the constructiveness of the placement remain stable over time: quality of supervision; optimal and relevant learning opportunities; and placement allocation relevant to year level of study. This evaluation, conducted over five years, demonstrates that the factors impacting engagement with learning must be considered in the design and development of placement programmes to ensure that learning opportunities for students are maximised.

## Figures and Tables

**Table 1:** University of Tasmania Nursing Student Placements.

UTAS Campus Region (State)	MMM Classification	Placement Type Available
Hobart (Tasmania)	2 (Regional)	Urban and rural
Launceston (Tasmania)	2 (Regional)	Urban and rural
Northwest (Tasmania)	3 (Large Rural)	Rural
Sydney (New South Wales)	1 (Major city)	Urban and rural

**Table 2:** Survey responses by survey year, student year, and placement regions.

	2017	2018	2019	2020	2021	Totals
Survey responses *	551 (18%)	582 (19%)	592 (17%)	529 (17%)	827 (27%)	3081
Urban placements	421	421	446	404	656	2348 (76%)
Rural placements	130	161	146	125	171	733 (24%)
1-year students	115	139	120	220	156	750 (24%)
2-year students	294	203	254	147	368	1266 (41%)
3-year students	142	240	218	162	303	1065 (35%)
Hobart placements	147	205	233	191	259	1035 (34%)
Launceston placements	112	118	84	100	120	534 (17%)
Sydney placements	162	98	129	113	277	779 (25%)
Rural TAS placements	126	151	130	123	161	691 (22%)
Rural NSW placements	4	10	16	2	10	42 (1%)

* Response rates calculated as percentage of all data for analysis period. It is not calculated on response rate for total students attending placement each year, as this information was not made available to the research team.

**Table 3:** The coding variables and categories for each survey question.

Survey Q1	Survey Q2	Survey Q3
*What helped learning*	*What hindered learning*	*Suggested improvements*
Category 1—Overall placement experience		
Nothing helped learning	Nothing hindered learning	Had a constructive placement
Category 2—Supervision quality		
Praise for university funded facilitators	Criticisms of university funded facilitators	Praise or criticisms of university funded facilitators
Praise for placement preceptors, supervisors, and staff	Criticisms of placement preceptors, supervisors, and staff	Praise or criticisms of placement preceptors, supervisors, and staff
Category 3—Skills development during placement		
Developed clinical skills Developed interpersonal skills Optimal placement facilities and infrastructure	Limited opportunities to develop clinical skills Scope of practice restrictions Experienced difficulties learning work tasks	Developed clinical skills Need more opportunities to develop clinical skills Need acute settings to develop skills Need more information about scope of practice

**Table 4:** Overall placement experience by group, as percentages.

Survey Question	What Helped Learning?	What Hindered Learning?	Suggested Improvements
Coding Variable	Nothing Helped Learning	Nothing Hindered Learning	Experienced a Constructive Placement
All responses	2%	19%	15%
Urban placements	2%	20%	15%
Rural placements	2%	19%	17%
First-year students	1%	21%	18%
Second-year students	2%	17%	15%
Third-year students	3%	21%	14%

**Table 5:** Student narratives on overall placement experience.

Survey Question 1	Survey Question 2	Survey Question 3
Nothing Helped Learning	Nothing Hindered Learning	Had a Constructive Placement
It is difficult for me to reflect with anything positive about this experience. Any positives have been far over shadowed by the negatives (Third-year student, Sydney 2017)	I enjoyed my PEP. I don’t think anything hindered my learning (Second-year student, Sydney 2021)	It was the best experience I have in two weeks and feeling proud to choosing the right course and looking forward to having other PEP (First-year student, Sydney 2017)
I found this placement to be very unsupportive and lacked direction; I am unable to identify aspects that helped my learning (Second-year student, Hobart 2017)	Nothing, it was a great prac (Third-year student, rural TAS 2020)	The whole PEP placement has been an excellent learning experience (Second-year student, rural NSW 2018)
There [was] no aspect of this placement that allow me to learn (Third-year student, rural NSW 2018)	None. I enjoyed the PEP because it strengthened and consolidated my learning. I am looking forward to another PEP opportunity next year (First-year student, Hobart 2019)	I was extremely fortunate to have a wonderful placement (Third-year student, Hobart 2019)

**Table 6:** Responses for placement supervision, as percentage of each student group.

	Survey Question 1		Survey Question 2	
	Praise for UFFs	Praise for Placement Supervisors	Criticisms of UFFs	Criticisms of Placement Supervisors
All responses	20%	53%	7%	31%
Urban placements	23%	52%	7%	31%
Rural placements	11%	55%	5%	29%
First-year placements	14%	53%	3%	31%
Second-year placements	19%	50%	7%	31%
Third-year placements	26%	55%	9%	30%

**Table 7:** Exemplars of student narratives about placement supervision.

Survey Question 1	Survey Question 2	Survey Question 1	Survey Question 2
Praise for UFFs	Supervision Criticisms of UFFs	Praise for Placement Supervision	Supervision Criticisms of Placement Supervision
Excellent facilitator attitude toward me as a person (Third-year student, Launceston 2017)	No facilitator available (Third-year student, rural TAS 2019)	Working with aged care staff (First-year student, Hobart 2020)	Unwelcoming buddy RN at times (Third-year student, Sydney 2017)
My facilitators did a phenomenal job with engaging with all of their students and giving myself and other students numerous opportunities to build upon prior learning and practices (Third-year student, Hobart 2019)	Feeling disempowered by facilitator at times resulting in tears with me as a student (Second-year student, Launceston 2018)	Supportive staff members who are welcoming to teach students (Second-year student, rural TAS 2019)	It felt like the nurses didn’t know that there would be students, cause every morning the nurses would be confused about why we were there, which made me feel like I was in the way of them doing their work, so I felt like I couldn’t ask as many questions or even ask to follow them around most days (Second-year student, Hobart 2020)
My facilitator was incredibly helpful with clinical, placement and personal issue throughout the two weeks. She actively sought to help educate whilst allowing us to continue our jobs within the ward (First-year student, Sydney 2021)	My university facilitator from my first week of PEP … was totally unsupportive of me, in fact very rude (Third-year student, Sydney 2021)	Great support from my facilitator and preceptors to work through placement (Third-year student, Launceston 2017)	Given tasks to do not being able to complete them before being told to do or be somewhere else (Second-year student, rural TAS 2018)

**Table 8:** Percentage of responses for skills development during placement.

	All Responses	Urban Placements	Rural Placements	First-Year Placements	Second-Year Placements	Third-Year Placements
Developed clinical skills	43%	42%	45%	50%	44%	36%
Developed interpersonal skills	26%	28%	21%	31%	24%	26%
Limited opportunities to develop clinical skills	24%	23%	26%	22%	26%	22%
Need more opportunities to develop skills	8%	8%	8%	5%	9%	9%
Scope of practice restricted skills development	7%	7%	6%	18%	4%	2%
Acute settings necessary to develop skills	4%	4%	4%	1%	6%	4%

**Table 9:** Exemplars of student’s comments about skills development during placements.

Developed Clinical Skills During Placement	Developed Interpersonal Skills During Placement	Lack of Opportunities to Develop Clinical Skills
Acute setting gave high exposure and opportunity to develop my practice (Third-year student, Hobart 2018)	Further developed my communication skills with doctors and other health professionals (Second-year student, Hobart 2019)	This placement is not suitable for second year students as it does not provide any learning opportunities or the chance to learn any new skills (Second-year student, rural TAS 2018)
Getting confidence on taking vital signs, including Manual Blood Pressure and Blood Glucose Level (First-year student, Sydney 2017)	Figuring out my strength and weakness, improving communication skills with colleagues and patients (First-year student, Launceston 2018)	Being sent to perform observations when the RN is doing something more interesting that is within my scope of practice and learning—having the AINs perform most of the ADL work, meaning there are less opportunities for me to participate in them (First-year student, Sydney 2017)
Medication administration (Second-year student, rural TAS 2019)	Learning to work cooperatively and collaboratively with others and respecting each other’s opinions and views. I’ve enjoyed exploring my beliefs and views and being able to have healthy conversations with my preceptors regarding those things. Particularly regarding palliation (Third-year student, rural TAS 2017)	There were a few times when the CFHN declined me attending a home visit with them without giving a reason. Very frustrating for me and it felt like I was wasting my time (Third-year student, rural NSW 2021)
**Scope of Practice Restrictions Limited Skills Development**	**Need More Opportunities to Develop Clinical Skills**	**Acute settings Needed to Develop Clinical Skills**
Limited scope of practice (First-year student, Sydney 2021)	This allocation is inappropriate for a final placement, there is almost NIL clinical consolidation and there is a LOT of down time (Third-year student, rural TAS 2019)	Psychiatry is very good as a first or second-year placement, however, I would not recommend this as a third-year placement as there are little to no nursing medical skills which are practiced (Third-year student, Hobart 2018)
Limits to practice due to my small scope of practice as a first-year student (First-year student, rural TAS 2020)	Inservice regarding common patient clinical presentations and operations prior on-floor activities would be beneficial so that students have fundamental understandings of how wards operate (Third-year student, Sydney 2021)	I understand that the prison is an accredited health facility, however, I do not believe that it is a good place for placement. There is minimal contact with “patients” very little opportunity to practice clinical skills and mental health learning is limited (Second-year student, Hobart 2019)
Misunderstanding of my practice ability: not all nurses were aware of what we can and can’t do (Second-year student, Hobart 2020)	I think having a specific preceptor to look after students is really important. I am reasonably confident and work well with most people but some days I have wandered aimlessly and no one has offered to show me anything and often decline my offers of help (First-year student, Hobart 2021)	As far as possible, nursing students should be sent to hospitals (not nursing home or aged care facilities) for their PEP as a wide variety of skills can be observed, learned and practiced in hospital setting. The scope of skills that can be learnt in nursing homes and aged care facilities is too limited (First-year student, Sydney 2017)

## Data Availability

The data presented in this study are available on request from the corresponding author due to ethical approval.

## Abbreviations

The following abbreviations are used in this manuscript:

GenAI	Generative Artificial Intelligence
MMM	Modified Monash Model
PEP	Professional experience placements
RHMT	Rural Health Multidisciplinary Training
UFF	University Funded Facilitators
UTAS	University of Tasmania
